# Early Gut Microbiome–Short-Chain Fatty Acid Axis Disruption May Be Associated with Delayed Recovery in Critically Ill Children

**DOI:** 10.3390/nu18101543

**Published:** 2026-05-13

**Authors:** Yoon Kyung Cho, Kyeong Hun Lee, Hyun Mi Kang, In Kyung Lee

**Affiliations:** 1 Department of Pediatrics, Seoul St. Mary’s Hospital, Seoul 06591, Republic of Korea; 2 Department of Pediatrics, College of Medicine, The Catholic University of Korea, Seoul 06591, Republic of Korea

**Keywords:** pediatric intensive care unit, gut microbiome, short-chain fatty acids, butyrate, critical illness

## Abstract

**Background:** The gut microbiome contributes to immune–metabolic homeostasis through microbial-derived metabolites such as short-chain fatty acids (SCFAs). However, whether early disruption of the gut microbiome–SCFA axis identifies impaired clinical recovery in pediatric intensive care unit (PICU) patients remains unclear. Biological markers reflecting the recovery trajectory beyond conventional severity scores remain poorly characterized in pediatric critical illness. We therefore investigated whether early microbiome disruption and fecal SCFA profiles are associated with recovery trajectory in critically ill children. **Methods:** In this prospective observational study (N = 26), fecal samples were collected within 5 days of PICU admission. Microbial diversity was assessed using 16S rRNA gene sequencing (Shannon index), and fecal SCFAs were quantified using targeted metabolomics. Disease severity was assessed using the Pediatric Index of Mortality 3 (PIM3). The primary outcome was PICU length of stay (LOS) as a pragmatic indicator of metabolic and functional recovery trajectory in critically ill children. **Results:** Younger age and higher disease severity showed a trend toward reduced microbial diversity (β = 0.066, *p* = 0.089, and β = −0.054, *p* = 0.089). Early loss of gut microbial diversity was associated with reduced fecal butyric acid concentrations (r = 0.440, *p* = 0.024). Importantly, lower microbial diversity in the early sampling window showed a significant inverse correlation with PICU LOS (ρ = −0.428, *p* = 0.029), whereas fecal butyric acid alone was not directly associated with LOS (*p* = 0.321). In multivariable regression models adjusting for age, disease severity, and clinical exposures, microbial diversity showed a consistent inverse association with PICU LOS, although statistical significance was not reached. **Conclusions:** Early disruption of the gut microbiome–SCFA axis, characterized by reduced microbial diversity and lower fecal butyrate, showed trend-level associations with delayed clinical recovery in this pilot cohort. Gut microbial ecosystem integrity may serve as a biologically relevant marker of recovery trajectory beyond conventional severity scoring.

## 1. Introduction

Severity scoring systems are widely used in pediatric intensive care units (PICUs) to estimate mortality and support early clinical decision-making [1]. In critically ill children, survival alone does not equate to recovery, and substantial heterogeneity exists in the trajectory of clinical improvement even among patients with comparable initial severity [2,3]. Identifying biological markers that reflect physiological recovery and metabolic resilience, particularly those reflecting nutritional homeostasis during critical illness, remains an unmet need in pediatric critical care.

The gut microbiome is increasingly recognized as a central regulator of immune–metabolic homeostasis and host resilience during critical illness. Through the production of microbial-derived metabolites such as short-chain fatty acids (SCFAs) [4], the gut microbiome modulates mucosal barrier integrity, immune tolerance, and systemic inflammatory responses [5]. Among these metabolites, butyric acid plays a pivotal role in systemic immune responses by promoting regulatory T-cell differentiation, enhancing intestinal epithelial barrier function, and maintaining intestinal immune homeostasis [6,7]. Because SCFA production depends on microbial fermentation of dietary substrates, disruption of this axis may reflect impaired host–microbiome nutritional interactions during critical illness. Accordingly, the gut microbiome can be viewed as a metabolic organ linking nutritional substrate availability with immune–metabolic homeostasis. This framework provides a biologically plausible rationale for investigating microbiome-derived metabolites as markers of recovery trajectories in critical illness. Consistent with this concept, alterations of the gut microbiome–SCFA axis have been linked to adverse outcomes and nosocomial infections in adult critical illness [8,9,10]. However, data in pediatric populations remain limited, despite the distinct immunologic and metabolic characteristics of children [11].

While gut dysbiosis is common in critical illness [12,13,14], its clinical implications in pediatric recovery trajectories remain poorly defined. Importantly, it is unknown whether ecosystem-level microbial disruption provides information beyond conventional severity scores in predicting recovery trajectories. We therefore conducted a prospective observational pilot study in a PICU to examine the relationship between early gut microbial diversity, microbiome-derived butyric acid, and subsequent clinical recovery. We hypothesized that early disruption of the gut microbiome–SCFA axis may be associated with a biologically distinct delayed recovery phenotype that conventional severity scores fail to capture.

## 2. Methods

### 2.1. Study Design

This prospective observational study was conducted at a tertiary PICU of Seoul St. Mary’s Hospital. We screened all children admitted to the PICU between April 2025 and October 2025 for eligibility. This pilot enrollment period was determined by the feasibility of prospective sample collection within the funded study timeframe. Patients were enrolled if they were older than 1 month and younger than 18 years. To ensure the integrity of the gut microbiome analysis, we excluded patients with a history of gastrointestinal surgery affecting the colon or pre-existing inflammatory bowel disease. Furthermore, patients were excluded if a fecal sample could not be obtained within the designated sampling window. The Institutional Review Board of Seoul St. Mary’s Hospital approved the study protocol (IRB number 2024-2121-0003), and we obtained written informed consent from the parents or legal guardians of all participants.

### 2.2. Sample Collection

To capture the early gut microbial landscape during the acute phase of illness, fecal samples were collected within the first five days of PICU admission. We collected samples using sterile techniques from diapers or bedpans, immediately transferred them to an ice-filled container, and stored them at −80 °C to preserve microbial and metabolic integrity. For patients with multiple samples, only the first stool specimen was utilized for analysis. This early window was selected to minimize confounding by prolonged ICU-related interventions and to capture microbiome alterations reflective of the initial physiological insult. Samples underwent a single freeze–thaw cycle prior to DNA extraction.

### 2.3. Microbial DNA Extraction and 16S rRNA Sequencing

Microbial DNA was extracted from fecal samples using the QIAamp DNA Stool Mini Kit (Qiagen, Hilden, Germany) according to the manufacturer’s instructions. To characterize the gut microbiota, the V3–V4 hypervariable regions of the bacterial 16S rRNA gene were amplified using specific primers: 341F (5′-CCTACGGGNGGCWGCAG-3′) and 805R (5′-GACTACHVGGGTATCTAATCC-3′). Sequencing was performed on the Illumina MiSeq platform (Illumina Inc., San Diego, CA, USA), generating 250 bp paired-end reads.

Raw paired-end reads were processed using the CD-HIT-OTU pipeline. Briefly, short or low-quality reads were filtered, ambiguous bases were removed, and primer/adaptor tails were trimmed. Duplicate reads were clustered at 100% identity using CD-HIT-DUP to reduce redundancy. Chimeric sequences were detected and removed, and representative non-chimeric sequences were clustered into operational taxonomic units (OTUs) at 97% sequence similarity. Taxonomic assignment was performed using the SILVA reference database based on representative OTU sequences.

To account for differences in sequencing depth across samples (total reads = 1,360,524; 42,161–62,147 reads per sample), samples were rarefied to an even depth of 42,000 reads per sample for alpha- and beta-diversity analyses. Rarefaction depth was selected based on the minimum sequencing depth across samples while maintaining Good’s coverage >99% in all samples, ensuring adequate representation of community diversity. Alpha diversity was assessed using the Shannon index. Beta diversity was calculated using Bray–Curtis dissimilarity and visualized by multidimensional scaling (MDS). No negative extraction controls were included in this pilot study. Although fecal samples are generally high in biomass, low-level reagent contamination cannot be excluded. Therefore, these microbiome findings should be interpreted as hypothesis-generating.

### 2.4. Targeted Metabolomic Analysis of SCFAs

Fecal concentrations of SCFAs, including acetic acid, propionic acid, and butyric acid, were quantified using gas chromatography (GC). Fecal samples (approximately 100 mg) were homogenized in distilled water and acidified with 25% metaphosphoric acid. After centrifugation, the supernatant was filtered and analyzed using the Agilent 7890B GC system (Agilent Technologies, Santa Clara, CA, USA) equipped with a flame ionization detector (FID) and a DB-FFAP column (30 m × 0.25 mm × 0.25 μm; Agilent Technologies, Santa Clara, CA, USA).

The oven temperature was programmed to increase from 100 °C (held for 2 min) to 240 °C at a rate of 10 °C/min. Nitrogen was used as the carrier gas at a constant flow rate. SCFAs were identified and quantified by comparing their retention times and peak areas with those of external standards (acetic, propionic, and butyric acids). Results were expressed as µmol per gram (µmol/g) of wet feces. Calibration curves were generated using external SCFA standards, and measurements were performed in duplicate when sample volume permitted.

### 2.5. Clinical Data Collection

We prospectively collected demographic and clinical data from electronic medical records, including age, sex, admission diagnosis, underlying comorbidities, and immunocompromised status. Admission reasons were categorized into major clinical groups (shock, respiratory, neurological, kidney injury, postoperative care, and others) to provide a clinical context for microbiome interpretation. Immunocompromised status was defined as receipt of chemotherapy or other immunosuppressive therapy, primary immunodeficiency, solid organ or hematopoietic stem cell transplantation, or prolonged systemic corticosteroid use. Infection at admission was defined by a clinician-diagnosed infection with corresponding antimicrobial treatment initiated within 24 h of PICU admission. Early nutrition variables (enteral feeding/TPN on admission day) were extracted from medication and nutrition order records. We also recorded exposures to antibiotics and proton pump inhibitors (PPIs) prior to fecal sampling.

Baseline disease severity was quantified using the Pediatric Index of Mortality 3 (PIM3) score calculated at the time of admission [15]. The primary outcome was PICU length of stay (LOS), serving as a pragmatic surrogate for the clinical recovery trajectory and resource utilization [16,17,18].

### 2.6. Statistical Analysis

Continuous variables are presented as medians (interquartile range), while categorical data are shown as frequencies (%). To explore the interplay between microbial diversity, butyric acid, and clinical metrics, normality was assessed using the Shapiro–Wilk test, and Pearson or Spearman correlations were applied accordingly. The influence of disease severity (PIM3) on microbial diversity was evaluated using linear regression. To isolate the independent effect of clinical factors on the microbiome, we constructed multivariate regression models adjusting for age, severity scores, and medication exposure (antibiotics and PPIs). PICU LOS was log-transformed due to a right-skewed distribution. Detailed nutritional composition data were not consistently available and therefore were not included in multivariable analyses. A two-sided *p*-value < 0.05 was considered statistically significant. As a sensitivity analysis, ventilator-free days at 28 days (VFD28) were evaluated as an alternative outcome. VFD28 was defined as 28 minus the number of days of invasive mechanical ventilation; patients who died within 28 days were assigned a VFD28 of 0. Given the pilot sample size, models were kept parsimonious to avoid overfitting. Post hoc power analysis based on the observed effect size (Spearman ρ = −0.428) using Fisher’s z-transformation indicated that a sample size of 41 would be required to achieve 80% power at a two-sided α = 0.05. The current pilot cohort (N = 26) achieved an estimated power of 59.2%, consistent with its hypothesis-generating intent. All statistical analyses were conducted using Stata version 19 (StataCorp, College Station, TX, USA).

## 3. Results

### 3.1. Baseline Characteristics

Twenty-six critically ill children were enrolled (median age 2.0 years [IQR 1.0–12.0]; 53.8% male). Respiratory failure was the most common admission reason (30.8%). Median PIM3 at admission was 4.4 (IQR 1.8–8.5), and 46.2% were immunocompromised. Clinician-diagnosed infection at admission was present in 46.2%, and 42.3% received PPIs prior to sampling. Median Shannon diversity was 2.4 (IQR 1.7–2.8), and median fecal butyric acid concentration was 8.0 μmol/g (IQR 4.2–13.6). Median PICU LOS was 12.5 days (IQR 6.0–52.0). Baseline characteristics are summarized in Table 1.

### 3.2. Clinical Determinants of Early Gut Dysbiosis

**Figure 1:**
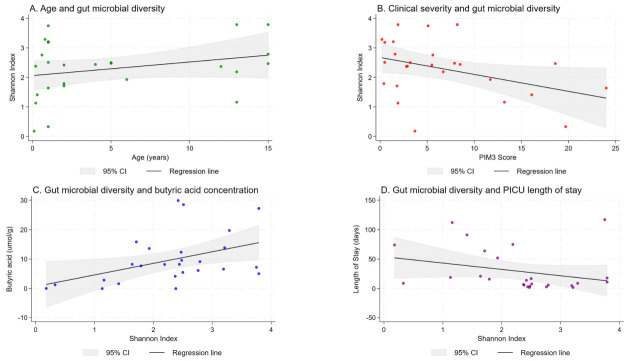
The gut microbial–metabolite–clinical outcome axis in pediatric intensive care unit patients. Each dot represents an individual patient. (**A**) Trend toward an association between age and microbial diversity. (**B**) Trend toward an inverse association between disease severity (PIM3 score) and microbial diversity. (**C**) Positive association between Shannon diversity index and fecal butyric acid concentration. (**D**) Inverse association between microbial diversity and length of stay in the PICU.

**Figure 2:**
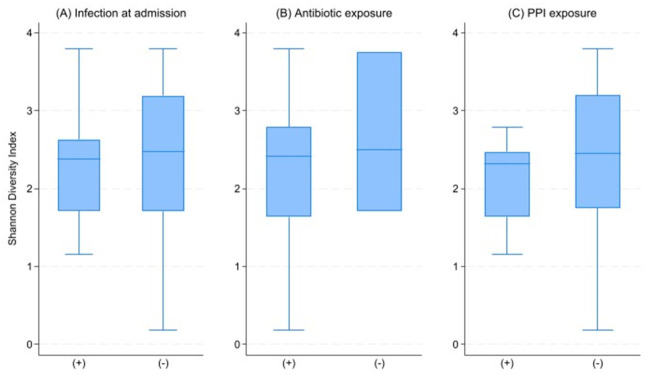
Gut microbial diversity according to clinical exposures at PICU admission. Boxplots show Shannon diversity index stratified by (**A**) infection status at admission, (**B**) antibiotic exposure prior to sampling, and (**C**) proton pump inhibitor (PPI) exposure. No significant differences in alpha diversity were observed between exposure groups, suggesting that early microbial diversity may be more closely related to underlying host factors and illness severity than to specific medication exposures in this early sampling window.

We examined clinical factors associated with gut microbial diversity measured in the early sampling window (within five days of PICU admission). Younger age and higher admission severity (PIM3) showed trend-level associations with lower Shannon diversity (Figure 1A,B). In multivariable linear regression, both PIM3 score (β = −0.054, *p* = 0.089) and age (β = 0.066, *p* = 0.089) demonstrated consistent trend-level associations with Shannon diversity, independent of antibiotic or PPI exposure (Table 2). Categorical clinical exposures, including infection status at admission, antibiotic exposure prior to sampling, and PPI exposure, were not associated with Shannon diversity at this time point (Figure 2; Supplementary Table S1).

In correlation analyses (Table 3), higher PIM3 tended to be associated with lower Shannon diversity (Spearman ρ = −0.342, *p* = 0.089). Lower Shannon diversity was associated with lower fecal butyric acid (Spearman ρ = 0.412, *p* = 0.035) and longer PICU LOS (Spearman ρ = −0.428, *p* = 0.029).

### 3.3. Microbial Community Structure and Taxonomic Shifts

**Figure 3:**
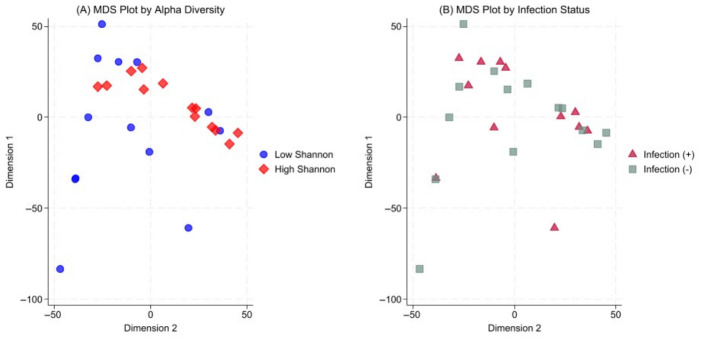
Multidimensional scaling (MDS) plot of gut microbiome beta diversity in pediatric intensive care unit patients. (**A**) Samples stratified by alpha diversity level (Shannon index, median split) demonstrate structural heterogeneity of the microbial ecosystem. Taxonomic differences underlying the observed beta-diversity pattern include higher relative abundances of *Bacteroidota* (*p* = 0.043) and *Actinomycetota* (*p* = 0.048) in the high-diversity group. (**B**) When colored by infection status at admission, samples show no distinct separation, suggesting that early beta-diversity patterns were not driven by infection status alone in this cohort.

Beta-diversity analysis demonstrated marked inter-individual heterogeneity in gut community structure in the early sampling window (Figure 3A). When stratified by Shannon diversity (median split), the high-diversity group exhibited higher relative abundances of *Bacteroidota* and *Actinomycetota* compared with the low-diversity group (*p* = 0.043 and *p* = 0.048, respectively), whereas *Pseudomonadota* tended to be higher in the low-diversity group (*p* = 0.086) (Supplementary Table S2). When samples were colored by infection status at admission, infected and non-infected patients remained intermingled without distinct separation (Figure 3B).

### 3.4. Association Between Microbial Diversity, SCFAs, and Recovery Trajectory

Shannon diversity was positively associated with fecal butyric acid (Spearman ρ = 0.412, *p* = 0.035; Pearson r = 0.440, *p* = 0.024; Figure 1C). No significant correlations were observed between Shannon diversity and acetic acid or propionic acid (*p* = 0.806 and *p* = 0.101, respectively).

Lower Shannon diversity measured early after PICU admission was associated with longer PICU LOS (Spearman ρ = −0.428, *p* = 0.029; Figure 1D). In contrast, fecal butyric acid concentration alone was not associated with LOS (*p* = 0.321; Table 3), nor were acetic or propionic acids (*p* = 0.590 and *p* = 0.340).

In multivariable linear regression models with log-transformed LOS, Shannon diversity showed a consistent inverse association with log(LOS) across models adjusting for age and PIM3 (β ≈ −0.55; *p* ≈ 0.08–0.09). Additional adjustment for immunosuppression or infection status did not materially change the effect estimate (Table 4).

VFD28 was considered as an alternative physiology-driven outcome. However, 10 of 26 patients (38.5%) did not require invasive mechanical ventilation, resulting in a ceiling effect (VFD28 = 28) that limited the discriminatory capacity of this metric in our heterogeneous cohort. A sensitivity analysis using VFD28 showed a directionally consistent but non-significant association with Shannon diversity (ρ = 0.302, *p* = 0.134). Furthermore, several patients demonstrated prolonged PICU LOS despite early ventilator liberation, reflecting ongoing organ dysfunction beyond respiratory recovery. These observations support the use of LOS as a more comprehensive recovery surrogate in this pilot cohort.

To assess whether enteral nutritional substrate availability affected fecal butyrate concentration, we compared butyric acid levels according to enteral nutrition (EN) and total parenteral nutrition (TPN) status on the admission day. No significant differences in fecal butyric acid were observed between patients who received EN and those who did not (median 7.47 vs. 10.89 μmol/g, *p* = 0.455), nor between patients who received TPN and those who did not (median 4.52 vs. 8.70 μmol/g, *p* = 0.126). Similarly, Shannon diversity did not differ significantly by EN status (*p* = 0.374). These findings suggest that the observed variation in fecal butyrate concentrations was not primarily driven by acute differences in enteral substrate availability on the admission day, although detailed longitudinal nutritional intake data were unavailable.

## 4. Discussion

This prospective pilot study suggests that early disruption of the gut microbiome–SCFA axis may be associated with delayed recovery in critically ill children. Specifically, lower microbial ecosystem diversity measured early after PICU admission was associated with lower fecal butyrate levels and a longer PICU LOS, a pragmatic marker of recovery trajectory in settings where mortality is uncommon.

Prior pediatric studies have reported gut dysbiosis and metabolic perturbations in sepsis and critical illness, often by comparing specific disease groups with healthy controls [19]. Our study extends this literature by evaluating a heterogeneous PICU cohort and focusing on recovery-relevant endpoints rather than disease labeling. Importantly, we link an ecosystem-level measure (Shannon diversity) to both a functional readout (fecal butyrate) and a clinical trajectory (LOS), supporting the concept that microbiome integrity may reflect biological resilience not fully captured by conventional severity scores.

We observed that younger age and higher PIM3 showed trend-level associations with reduced microbial diversity. Although antibiotics and PPIs are established drivers of dysbiosis, these exposures were not associated with diversity in our early sampling window. This may indicate that, at the time of early PICU admission, acute physiological stress and underlying host vulnerability dominate ecosystem disruption, and iatrogenic exposures may exert larger effects later over time. Pediatric-specific factors, including the developing microbiome and immune system, may further increase susceptibility to rapid ecosystem disruption during critical illness.

Pediatric critical illness is characterized by distinct immunological and metabolic features that differ fundamentally from those of adults [20]. The developing immune system, evolving gut microbiome composition, and age-dependent metabolic demands may render children particularly vulnerable to early microbial ecosystem disruption during critical illness [21,22]. Unlike adults, in whom baseline microbiome stability may partially buffer acute physiological stress, children may experience a more rapid loss of microbial diversity and functional redundancy. This pediatric-specific vulnerability underscores the importance of early microbiome assessment and may partly explain the strong association observed between early microbial disruption and delayed clinical recovery in our cohort.

A key finding was that microbial diversity, rather than any single SCFA, showed the most consistent association with LOS. Although butyric acid is a well-established immunomodulatory metabolite essential for maintaining mucosal barrier integrity and immune tolerance [23], its direct association with PICU LOS did not reach statistical significance in this pilot cohort. This pattern suggests that recovery may depend more on ecosystem-level functional redundancy and stability than on the absolute concentration of one metabolite measured at a single time point. Diversity may integrate multiple features of the microbial ecosystem, including taxonomic richness, balance, and resilience, that collectively determine metabolic capacity and host–microbe interactions during acute stress.

To further interpret why ecosystem-level diversity was more closely related to recovery rather than individual metabolites, it is useful to conceptualize the gut microbiota as a functional metabolic organ during critical illness. The significant positive correlation between the Shannon index and fecal butyric acid levels (r = 0.440, *p* = 0.024) suggests that loss of microbial diversity may impair the functional capacity of the gut ecosystem. Butyric acid, a major SCFA, is well established as a key energy source for colonocytes and a regulator of intestinal barrier integrity and immune homeostasis [7]. In critical illness, depletion of butyrate-producing taxa has been associated with compromised epithelial barrier function and altered host-microbe interactions, which may facilitate systemic inflammatory signaling [24]. Although we did not directly assess intestinal permeability or immune cell function, the observed reduction in fecal butyric acid may plausibly reflect diminished microbiome-mediated support of host resilience. The functional impairment of the gut microbial ecosystem may contribute to a delayed clinical recovery trajectory in critically ill children. The absence of a direct association between fecal butyric acid levels and LOS may reflect the complexity of microbiome-derived metabolic networks. Ecosystem-level diversity likely captures functional redundancy and resilience beyond individual metabolite concentrations.

Beta-diversity analyses revealed marked inter-individual heterogeneity in gut microbial community structure at PICU admission. Lower alpha diversity was accompanied by relative depletion of commensal-associated phyla, consistent with previously reported dysbiosis patterns in critical illness. Infection status alone did not clearly separate community structure, suggesting that early microbiome disruption may reflect broader host physiological stress, although no distinct clustering was observed.

LOS in the PICU represents a pragmatic composite outcome reflecting the pace of clinical recovery, resolution of organ dysfunction, and readiness for discharge. While LOS is influenced by multiple non-biological factors such as organizational practices and discharge logistics [25], it remains a commonly used surrogate for recovery trajectory in pediatric critical care, particularly in studies where mortality is infrequent [16,18]. It may act as an indicator reflecting recovery pace, organ function stabilization, and readiness for clinical transition in pediatric critical care settings [18]. Importantly, our findings do not imply that gut microbiome disruption directly determines LOS, but rather that early microbial ecosystem status is associated with the subsequent recovery course. Conventional severity scores primarily capture acute physiological derangement and mortality risk [15], whereas microbial ecosystem integrity may reflect biological resilience and metabolic recovery capacity not captured by traditional scoring systems. Future studies incorporating longitudinal immune and metabolic markers will be required to disentangle causal pathways.

Early nutritional exposure may be relevant to interpreting microbiome-derived SCFA output in critically ill children, although this study was not designed to test nutritional interventions. In our cohort, a substantial proportion of patients received early enteral nutrition, and some required TPN (Table 1). Because SCFA production depends on microbial fermentation of available substrates [26], variation in early feeding route and substrate delivery could influence microbial metabolic activity and functional readouts such as fecal butyrate [27]. We did not have sufficient sample size or granular nutrition data, such as timing, composition, and fiber content, to model these effects, but future studies integrating detailed nutritional metrics with longitudinal microbiome–metabolite profiling are warranted.

Although microbiome-targeted interventions were not evaluated in this study, our findings suggest that early assessment of gut microbial ecosystem integrity may help identify children at risk for delayed recovery. Such identification could inform future strategies aimed at preserving microbial diversity during the earliest phase of critical illness, including cautious antimicrobial stewardship, early enteral nutrition, or microbiome-supportive care bundles [28,29,30]. Specific microbiome-targeted interventions warrant consideration in future studies. Probiotics and prebiotics have demonstrated safety signals in selected pediatric populations, though evidence in critically ill children remains limited and heterogeneous. Postbiotics, including exogenous butyrate supplementation, represent an emerging strategy that may bypass the need for intact microbial fermentation capacity, which is often compromised during critical illness. Future intervention trials integrating nutritional and microbiome-supportive strategies should carefully consider safety, optimal timing of initiation, and patient selection to maximize potential benefit in this vulnerable population. Importantly, these approaches emphasize ecosystem preservation rather than supplementation of isolated metabolites, aligning with the observation that global microbial diversity was more strongly associated with recovery trajectory than individual metabolic outputs.

**Figure 4:**
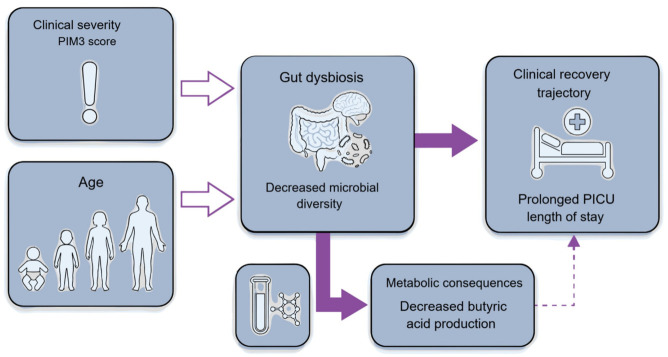
Proposed gut microbial-metabolite-clinical outcome axis in critically ill children. This conceptual diagram integrates the study findings, illustrating how age and early disease severity (PIM3) are associated with reduced microbial ecosystem integrity (decreased diversity and structural heterogeneity). This ecosystem disruption is linked to impaired production of microbiome-derived metabolites (butyric acid) and is associated with a prolonged clinical recovery trajectory, as reflected by PICU length of stay. Arrow styles indicate the relative direction and consistency of associations observed in this study: solid arrows represent stronger associations, open arrows indicate weaker associations, and thin arrows denote potential or indirect relationships.

We integrated these findings into a conceptual model of the microbiome–SCFA–clinical outcome axis (Figure 4). Unlike traditional severity scores that primarily estimate mortality risk, gut microbial diversity may capture aspects of biological recovery not reflected by conventional severity scores. Although certain associations in our multivariate models remained at the level of statistical trends, the consistent directional relationships observed between severity, diversity, and clinical outcomes suggest a biologically meaningful pathway that governs host resilience. This model positions early gut microbial status as a candidate marker that captures dimensions of biological recovery not currently reflected by conventional physiological scores.

As a single-center pilot study with a limited sample size, these findings should be interpreted as hypothesis-generating. Nevertheless, the prospective design and early sampling window allowed us to capture metabolically relevant signals during the acute phase of critical illness. The cross-sectional sampling design limits inference on temporal microbiome dynamics during critical illness. Stratified analyses by specific admission diagnoses or long-term post-PICU outcomes were not feasible in this cohort. All samples were collected within a predefined early window (within five days of PICU admission), although the exact sampling day relative to admission was not consistently recorded. Negative extraction controls were not included. While fecal samples typically contain high microbial biomass, low-level reagent contamination cannot be entirely excluded. In addition, fecal SCFA concentrations may vary according to sampling timing, enteral intake, stool consistency, and antibiotic exposure. We did not adjust for multiple comparisons given the pilot, hypothesis-generating design. Larger multicenter longitudinal studies are needed to validate these findings and to clarify whether microbiome- and nutrition-supportive strategies can actively accelerate recovery in critically ill children.

## 5. Conclusions

In conclusion, early disruption of the gut microbiome–SCFA landscape showed trend-level associations with delayed clinical recovery in this pilot cohort. Larger multicenter longitudinal studies are needed to validate these findings and to determine whether microbiome- and nutrition-supportive strategies can improve recovery trajectories in critically ill children.

## Figures and Tables

**Table 1:** Baseline Characteristics of the Study Cohort.

Characteristics	Study Cohort (N = 26)
Age (years)	2.0 [1.0–12.0]
Male sex, *n* (%)	14 (53.8%)
Admission reason	
Shock	6 (23.1%)
Respiratory failure	8 (30.8%)
Neurologic disease	6 (23.1%)
Acute kidney injury	3 (11.5%)
Post-operative	1 (3.8%)
Others	2 (7.7%)
Immunocompromised, *n* (%)	12 (46.2%)
PIM3 score	4.4 [1.8–8.5]
Infection at admission, *n* (%)	12 (46.2%)
PPI use at admission, *n* (%)	6 (23.1%)
Enteral feeding on admission day, *n* (%)	22 (84.6%)
Total parenteral nutrition on admission day, *n* (%)	10 (38.5%)
Shannon index	2.4 [1.7–2.8]
SCFAs	
Acetic acid, μmol/g	8.9 [3.0–27.9]
Propionic acid, μmol/g	5.2 [2.2–7.3]
Butyric acid, μmol/g	8.0 [4.2–13.6]
PICU length of stay, days	12.5 [6.0–52.0]
Mortality, *n* (%)	1 (3.8%)

Values are presented as median [interquartile range] or number (%), as appropriate.

**Table 2:** Multivariate linear regression analysis of clinical factors associated with gut microbiome diversity.

Dependent Variable	Independent Variable	Coefficient (β)	Std. Error	*p*-Value
Shannon index	PIM3 Score	−0.054	0.030	0.089
(R^2^ = 0.283)	PPI Use	−0.245	0.540	0.655
	Antibiotics Use	−0.316	0.557	0.577
	Age	0.066	0.037	0.089

**Table 3:** Correlation matrix of severity, microbiome, and clinical variables.

Variable	PIM3	Shannon Index	Butyric Acid	LOS
PIM3 Score	1.000			
Shannon index	−0.342 †	1.000		
Butyric acid, μmol/g	−0.154	0.440 *	1.000	
PICU length of stay, days	0.216	−0.428 *	−0.229	1.000

* *p* < 0.05, † trend toward significance (*p* < 0.1). Correlation coefficients are presented.

**Table 4:** Multivariable linear regression models for PICU length of stay.

Variable	Model 1		Model 2		Model 3	
	β (SE)	*p*	β (SE)	*p*	β (SE)	*p*
Shannon index	−0.537 (0.305)	0.092	−0.560 (0.304)	0.080	−0.550 (0.309)	0.090
Age	0.020 (0.048)	0.673	0.032 (0.049)	0.520	0.023 (0.048)	0.635
PIM3 score	−0.007 (0.042)	0.878	0.008 (0.044)	0.851	−0.010 (0.043)	0.820
Immunosuppression	–	–	0.590 (0.544)	0.290	–	–
Infection	–	–	–	–	−0.351 (0.501)	0.492
R^2^	0.142		0.187		0.161	

β: Regression coefficient, SE: Standard error. Dependent variable: log-transformed PICU length of stay. Model 1: Base. Model 2: Model 1 + Immune status. Model 3: Model 2 + Infection. –, variable not included in the model.

## Data Availability

The data presented in this study are available on request from the corresponding author due to privacy and ethical restrictions pertaining to patient data.

## Supplementary Materials

The following supporting information can be downloaded at: https://www.mdpi.com/article/10.3390/nu18101543/s1, Table S1: Comparative Analysis: Factors not directly altering diversity; Table S2: Comparison of fecal microbial composition at the phylum level.

## References

[1] Recher M., Leteurtre S., Canon V., Baudelet J.B., Lockhart M., Hubert H. (2022). Severity of illness and organ dysfunction scoring systems in pediatric critical care: The impacts on clinician’s practices and the future. Front. Pediatr..

[2] Woodruff A.G., Choong K. (2021). Long-term outcomes and the post-intensive care syndrome in critically ill children: A North American perspective. Children.

[3] Maddux A.B., Miller K.R., Sierra Y.L., Bennett T.D., Watson R.S., Spear M., Pyle L.L., Mourani P.M. (2024). Recovery trajectories in children requiring 3 or more days of invasive ventilation. Crit. Care Med..

[4] Tan J., McKenzie C., Potamitis M., Thorburn A.N., Mackay C.R., Macia L. (2014). The role of short-chain fatty acids in health and disease. Adv. Immunol..

[5] Belkaid Y., Hand T.W. (2014). Role of the microbiota in immunity and inflammation. Cell.

[6] Wang Z., Yu J., Liu Y., Gong J., Hu Z., Liu Z. (2025). Role of the microbiota–gut–lung axis in the pathogenesis of pulmonary disease in children and novel therapeutic strategies. Front. Immunol..

[7] Siddiqui M.T., Cresci G.A. (2021). The immunomodulatory functions of butyrate. J. Inflamm. Res..

[8] Sung J., Rajendraprasad S.S., Philbrick K.L., Bauer B.A., Gajic O., Shah A., Laudanski K., Bakken J.S., Skalski J., Karnatovskaia L.V. (2024). The human gut microbiome in critical illness: Disruptions, consequences, and therapeutic frontiers. J. Crit. Care.

[9] Oami T., Yamamoto A., Ishida S., Kondo K., Hata N., Oshima T. (2025). Critical Care Nutrition from a Metabolic Point of View: A Narrative Review. Nutrients.

[10] Schlechte J., Zucoloto A.Z., Yu I.-l., Doig C.J., Dunbar M.J., McCoy K.D., McDonald B. (2023). Dysbiosis of a microbiota–immune metasystem in critical illness is associated with nosocomial infections. Nat. Med..

[11] Chappell M.T., Kelly C., Rosenthal K.S. (2021). Why is a child not a miniadult for infections?. Infect. Dis. Clin. Pract..

[12] Rogers M.B., Firek B., Shi M., Yeh A., Brower-Sinning R., Aveson V., Kohl B.L., Fabio A., Carcillo J.A., Morowitz M.J. (2016). Disruption of the microbiota across multiple body sites in critically ill children. Microbiome.

[13] Yeh A., Rogers M.B., Firek B., Neal M.D., Zuckerbraun B.S., Morowitz M.J. (2016). Dysbiosis across multiple body sites in critically ill adult surgical patients. Shock.

[14] Xu J., Kong X., Li J., Mao H., Zhu Y., Zhu X., Xu Y. (2023). Pediatric intensive care unit treatment alters the diversity and composition of the gut microbiota and antimicrobial resistance gene expression in critically ill children. Front. Microbiol..

[15] Rahmatinejad Z., Rahmatinejad F., Sezavar M., Tohidinezhad F., Abu-Hanna A., Eslami S. (2022). Internal validation and evaluation of the predictive performance of models based on the PRISM-3 (Pediatric Risk of Mortality) and PIM-3 (Pediatric Index of Mortality) scoring systems for predicting mortality in Pediatric Intensive Care Units (PICUs). BMC Pediatr..

[16] Pollack M.M., Holubkov R., Reeder R., Dean J.M., Meert K.L., Berg R.A., Newth C.J., Berger J.T., Harrison R.E., Carcillo J. (2018). PICU length of stay: Factors associated with bed utilization and development of a benchmarking model. Pediatr. Crit. Care Med..

[17] Namachivayam P., Taylor A., Montague T., Moran K., Barrie J., Delzoppo C., Butt W. (2012). Long-stay children in intensive care: Long-term functional outcome and quality of life from a 20-yr institutional study. Pediatr. Crit. Care Med..

[18] Boerman G.H., Haspels H.N., De Hoog M., Joosten K.F. (2023). Characteristics of long-stay patients in a PICU and healthcare resource utilization after discharge. Crit. Care Explor..

[19] Sankar J., Thakral V., Bharadwaj K., Agarwal S., Kabra S.K., Lodha R., Rathore S. (2024). The Microbiome and Metabolome of the Gut of Children with Sepsis and Septic Shock. J. Intensive Care Med..

[20] Wheeler D.S., Wong H.R., Zingarelli B. (2011). Pediatric Sepsis–Part I:“Children are not small adults!”. Open Inflamm. J..

[21] Simon A.K., Hollander G.A., McMichael A. (2015). Evolution of the immune system in humans from infancy to old age. Proc. R. Soc. B.

[22] Veldscholte K., Joosten K., Jotterand Chaparro C. (2020). Energy expenditure in critically ill children. Pediatr. Med..

[23] Kullberg R.F., Wikki I., Haak B.W., Kauko A., Galenkamp H., Peters-Sengers H., Butler J.M., Havulinna A.S., Palmu J., McDonald D. (2024). Association between butyrate-producing gut bacteria and the risk of infectious disease hospitalisation: Results from two observational, population-based microbiome studies. Lancet Microbe.

[24] Dickson R.P. (2016). The microbiome and critical illness. Lancet Respir. Med..

[25] Temsah M.-H.A., Al-Eyadhy A.A., Al-Sohime F.M., Hassounah M.M., Almazyad M.A., Hasan G.M., Jamal A.A., Alhaboob A.A., Alabdulhafid M.A., Abouammoh N.A. (2020). Long-stay patients in pediatric intensive care units: Five-years, 2-points, cross-sectional study. Saudi Med. J..

[26] Mukhopadhya I., Louis P. (2025). Gut microbiota-derived short-chain fatty acids and their role in human health and disease. Nat. Rev. Microbiol..

[27] Oami T., Chihade D.B., Coopersmith C.M. (2019). The microbiome and nutrition in critical illness. Curr. Opin. Crit. Care.

[28] Fan L., Lee J.H. (2021). Enteral feeding and the microbiome in critically ill children: A narrative review. Transl. Pediatr..

[29] Neag M.A., Mitre A.O., Pomana I.G., Velescu M.A., Militaru C., Nagy G., Melincovici C.S. (2025). Host–Microbiome Interaction in the Intensive Care Unit. Diseases.

[30] Shimizu K., Ogura H., Oda J. (2025). Gut dysbiosis and its treatment in patients with critical illness. Acute Med. Surg..

