# Supplementary material for: Early Gut Microbiome–Short-Chain Fatty Acid Axis Disruption May Be Associated with Delayed Recovery in Critically Ill Children
Source: Nutrients. 2026 May 13;18(10):1543. doi: 10.3390/nu18101543 (PMC13210299; doi:10.3390/nu18101543)
Supplement: Supplementary file 1 [file nutrients-18-01543-s001.zip › Supplementary Table S1.pdf]

Supplementary Table S1. Comparative Analysis: Factors Not Directly Altering Diversity

Clinical Factor	Category	N	Shannon Index (median [IQR])	p-value*
Infection status	Positive	12	2.4 [1.7-2.6]	0.116
	Negative	14	2.5 [1.7-3.2]	
PPI exposure	Positive	6	2.3 [1.6-2.5]	1.000
	Negative	20	2.5 [1.8-3.2]	
Antibiotic exposure	Positive	23	2.4 [1.7-2.8]	0.539
	Negative	3	2.5 [2.1-3.1]	

*p-values were calculated using the Mann-Whitney U test.

Abbreviations: IQR: interquartile range; PPI: proton pump inhibitor.
