# Supplementary material for: Early Gut Microbiome–Short-Chain Fatty Acid Axis Disruption May Be Associated with Delayed Recovery in Critically Ill Children
Source: Nutrients. 2026 May 13;18(10):1543. doi: 10.3390/nu18101543 (PMC13210299; doi:10.3390/nu18101543)
Supplement: Supplementary file 1 [file nutrients-18-01543-s001.zip › Supplementary Table S2.pdf]

Supplementary Table S2. Comparison of Fecal Microbial Composition at the Phylum Level.

Phylum (Relative Abundance, %)	Low Shannon (n = 13)	High Shannon (n = 13)	p-value*
Bacteroidota	0.04 [0.02–28.95]	38.06 [14.39–53.27]	0.043
Actinomycetota	0.65 [0.32–5.79]	7.85 [4.65–10.24]	0.048
Pseudomonadota	25.66 [2.96–48.83]	6.76 [2.45–7.88]	0.086

*p-values were calculated using the Wilcoxon rank-sum test. Data are presented as median [interquartile range].
